# How to transition to reduced-meat diets that benefit people and the planet

**DOI:** 10.1016/j.scitotenv.2020.137208

**Published:** 2020-05-20

**Authors:** Niki A. Rust, Lucy Ridding, Caroline Ward, Beth Clark, Laura Kehoe, Manoj Dora, Mark J. Whittingham, Philip McGowan, Abhishek Chaudhary, Christian J. Reynolds, Chet Trivedy, Nicola West

**Affiliations:** a School of Natural and Environmental Sciences, Newcastle University, Newcastle NE1 7RU, UK; b UK Centre for Ecology and Hydrology, Wallingford, UK; c School of Earth and Environment, University of Leeds, Leeds, UK; d School of Natural and Environmental Sciences, Newcastle University, Newcastle NE1 7RU, UK; e University of Oxford, Oxford, OX1 2JD, UK; f The Nature Conservancy, Arlington County, Virginia, USA; g Operations Management, Brunel University, London, UK; h School of Natural and Environmental Sciences, Newcastle University, Newcastle NE1 7RU, UK; i Institute for Sustainability, Newcastle University, Newcastle NE1 7RU, UK; j Department of Civil Engineering, Indian Institute of Technology (IIT) Kanpur, India; k Department of Geography, University of Sheffield, Sheffield, UK; l National Health Service, London, UK; m West Midlands Deanery, UK

**Keywords:** Behaviour change, Carbon emissions, Healthy diets, Meat overconsumption, Planetary health, Sustainable diets

## Abstract

Overwhelming evidence shows that overconsumption of meat is bad for human and environmental health and that moving towards a more plant-based diet is more sustainable. For instance, replacing beef with beans in the US could free up 42% of US cropland and reduce greenhouse gas emissions by 334 mmt, accomplishing 75% of the 2020 carbon reduction target. We summarise the evidence on how overconsumption of meat affects social, environmental and economic sustainability. We highlight the social, environmental and economic effectiveness of a range of dietary interventions that have been tested to date. Because meat eating is embedded within complex cultural, economic, and political systems, dietary shifts to reduce overconsumption are unlikely to happen quickly and a suite of sustained, context-specific interventions is likely to work better than brief, one-dimensional approaches. We conclude with key actions needed by global leaders in politics, industry and the health sector that could help aide this dietary transformation to benefit people and the planet.

## Highlights

• Overconsumption of meat must decline to meet Sustainable Development Goals
• We review the range and effectiveness of interventions to reduce meat overconsumption
• Government and food supply chain buy-in are needed to transition to more sustainable diets

## Graphical abstract

**Figure:**
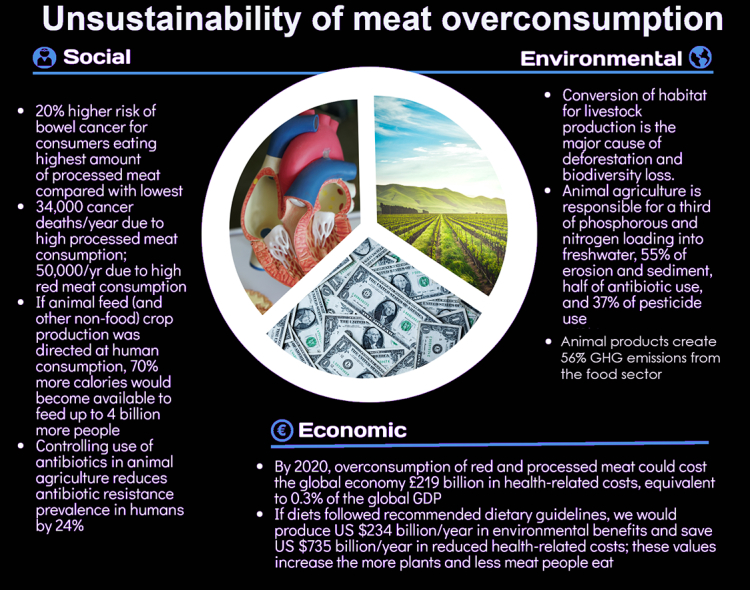

## 1 Introduction

Unsustainable food production and consumption negatively affect human and environmental health (Nyström et al., 2019). The most common cause of death globally is poor diet (Branca et al., 2019) causing 11 million deaths in 2017 (Afshin et al., 2019). Our food system is a leading driver of biodiversity loss (WWF, 2018) and contributes 19–29% of global greenhouse gas emissions (Vermeulen et al., 2012). This failing system severely limits our ability to achieve all of the United Nations Sustainable Development Goals (SDGs), so transitioning towards sustainable diets is urgently needed to ensure One Health objectives for people and the planet are achieved (Hawkes and Popkin, 2015).

The United Nations Food and Agricultural Organisation (FAO) defines sustainable diets as “*those diets with low environmental impacts which contribute to food and nutrition security and to healthy life for present and future generations. Sustainable diets are protective and respectful of biodiversity and ecosystems, culturally acceptable, accessible, economically fair and affordable; nutritionally adequate, safe and healthy; while optimizing natural and human resources”* (FAO, 2010). Thus, sustainable diets are those that holistically combine three core aspects: environmental, social (including health) and economic. To date, there has been a paucity of research that has addressed all three pillars of dietary sustainability together; instead, research has tended to focus on certain aspects of each pillar, such as reducing greenhouse gas emissions coupled with reducing overconsumption of animal protein (Friel et al., 2009; Westhoek et al., 2014; Clark and Tilman, 2017; National Academies of Sciences Engingeering and Medicine, 2019). For a diet to be considered truly “sustainable”, however, we argue that all three aspects of sustainability must be addressed together.

We focus on the overconsumption of meat, since on average, this has the greatest combined negative impact on environmental and human health (Godfray et al., 2018; Stoll-Kleemann and O'Riordan, 2015; Clark et al., n.d.) with ruminant meat production having an environmental impact (including land use, eutrophication, energy use, GHG emissions and acidification potential) 100 times greater than a plant-based diet (Clark and Tilman, 2017). Overconsumption of meat is where a person eats more than their recommended daily intake. In order to eat within our planetary boundaries (i.e. no net environmental damage), it has been estimated that we should consume no more than 98 g of red meat, 203 g of poultry and 196 g of fish per week (Willett et al., 2019). However, high-income countries are currently consuming double this (Stoll-Kleemann and O'Riordan, 2015), surpassing nutritional requirements (Sans and Combris, 2015), with many lower/middle income countries predicted to follow this trend over the coming decades (Tilman and Clark, 2014). For instance, red meat intake in North America, Latin America and Europe is 300–600% higher than daily recommended levels; poultry and egg intake in these regions also exceed recommended levels, whereas intake of fruits, vegetables and plant-sourced protein (e.g. from legumes) is approximately half of the recommended levels (Willett et al., 2019).

In this paper, we provide a summary of the social, economic and environmental costs of overconsumption of meat, followed by highlighting a suite of options for transitioning towards more sustainable diets with less meat and more plants. We go on to list key barriers, steps and global leadership needed to aid this system change.

### 1.1 Health and social consequences of overconsumption of meat

Whilst meat contains essential nutrients that can be important for human nutrition, such as B12, iron and calcium (Murphy and Allen, 2003), excessive consumption has been associated with adverse health outcomes. For example, overconsumption of red and processed meat has been linked to an increased risk of many non-communicable diseases (NCDs) such as coronary heart disease, type 2 diabetes, obesity and numerous cancers (Bouvard et al., 2015). People who consume red and processed meat four or more times per week have a 20% increased risk of colorectal cancer compared with those who consume it less than twice a week (Bradbury et al., 2019). Though meat is an important source of protein, in many high-income nations, average protein consumption surpasses dietary requirements, which contributes to numerous health problems, such as kidney and liver disorders, increased cancer and heart disease risks, and disorders of bone and calcium homeostasis (Delimaris, 2013). Although in this paper we argue for reducing meat consumption, it is worth noting that we would still be able to achieve satisfactory protein levels by cutting out meat entirely and instead obtaining our protein from plants and fungi (Macdiarmid et al., 2018).

The way in which animals are raised for human consumption can also affect our health. A heavy reliance on antibiotics in industrial-scale animal agriculture is contributing to antibiotic resistance in humans (Caffrey et al., 2017). Livestock act as reservoirs for pathogens that can infect people, especially where humans and farmed animals come into close contact (Jones et al., 2013). Meat is a source of various foodborne infections and intensive livestock production pollutes the air we breathe due to nitrogen compound and fine particulate matter emissions (Williams and Brent, 2017; Cambra-López et al., 2010; Tschofen et al., 2019). Compared with other occupations, including crop farming, livestock farmers are more likely to suffer from a variety of diseases, especially respiratory diseases (May et al., 2012). The human health implications of animal agriculture go well beyond those due to direct consumption.

The inefficiency of grain-fed livestock is a social problem, too. It is resource inefficient to feed human-edible grain to livestock, as energy is lost when converting human-edible grains to meat (Cassidy et al., 2013). Over one third of the grain we grow on earth is fed to livestock, with significant amounts of energy being lost along the way in the transfer of nutrients from plants to livestock and then on to humans (Cassidy et al., 2013). This exacerbates a food system that is already struggling to feed our global population. If the crop production currently used for animal feed (and other non-food uses) was instead directed at human consumption, it would create 70% more calories, which could feed up to 4 billion more people (West et al., 2014). Eating more meat than needed can also be regarded as a waste of food, as the people who consume too much meat require additional natural resources to be utilised to produce that meat, resulting in higher negative environmental impacts by producing more food.

Alongside overconsumption of meat, there are social consequences of unsustainable livestock production too (Hoekstra and Chapagain, 2006). Meat production, broadly, uses ~22% of global freshwater, of which beef tends to have the largest footprint, primarily from irrigating feed crops that are used in the grain-finishing stage (Hoekstra and Chapagain, 2006). For instance, if water is scarce, this may result in competition for water between humans and livestock (Schlink et al., 2010). Industrial-scale animal agriculture, driven primarily by the increasing demand for more and cheaper meat, can reduce social capital of rural communities (Thu, 1996). Large-scale operations can be disproportionately situated next to communities with a high density of low-income and people of colour (Wing et al., 2000). These large farms suffer from odour issues, which can affect the mental health of local communities (Schiffman et al., 1995). In Latin America, where large tracts of forest have been cut down to make way for soybean production (90% of which is used to feed livestock), local incomes have declined in some areas (Correia, 2019) and many smaller land owners have been priced out of the area or forced to sell their farms to large producers, with rural unemployment increasing (Burley, 2008).

### 1.2 Environmental consequences of overconsumption of meat

On top of the social concerns, rising demand for meat has created substantial environmental impacts. Globally, animal products provide only 18% of our calories but use 83% of our farmland and are responsible for 56% of GHG emissions from the food sector (Poore and Nemecek, 2018). Animal agriculture is also a leading cause of habitat destruction - such as deforestation in the Amazon - to raise livestock and grow livestock feed (Steinfeld et al., 2006). Cattle farming is currently directly responsible for 71% of Latin American deforestation, making it the single largest driver of deforestation across the region (De Sy et al., 2015), and resulting in an annual forest loss of 2.71 million hectares - ten times the amount of deforestation as palm oil (Henders et al., 2015).

Other ecosystem services are also degraded by meat production. Livestock production can contaminate freshwater supplies, mainly through nutrient use in feed production, as well as manure management in feedlots, barnyards, and other facilities. Freshwater pollution is a major contributor to dead zones in estuaries and coastal regions, of which there are now more than 400 globally (Diaz and Rosenberg, 2008). Unsustainable livestock production disproportionately contributes to the environmental cost of agriculture, through high resource use including water, land and soil, as well as being a key driver of biodiversity loss and greenhouse gas emissions (Steinfeld et al., 2006). Given the increasing demand and need for meat consumption in areas that do not currently consume enough to meet nutritional requirements, overconsumption of meat must decline to ensure that, globally, we are not placing additional burden on the earth's life support systems.

If each country was to adopt a sustainable diet (i.e. follow their country's recommended dietary guidelines, which results in Western nations reducing their meat consumption and increasing consumption of plants), this will reduce the global greenhouse gas (GHG) emissions by approximately 30% and reduce the freshwater withdrawals, nitrogen and phosphorus application by 10–15% while keeping the footprint of food production at a global level at the current level (Chaudhary and Krishna, 2019; Springmann et al., 2018c; Springmann et al., 2018a). Adoption of sustainable diets for countries that currently overconsume meat and under-consume plants will bring the food-related environmental footprints of each country below planetary boundaries (Willett et al., 2019; Chaudhary and Krishna, 2019).

### 1.3 Economic consequences of overconsumption of meat

It is estimated that overconsumption of red and processed meat will cost the global economy £219 billion in health-related costs in 2020, equivalent to 0.3% of the global GDP (McLachlan, 2018). Patients with illnesses related to meat overconsumption can incur financial costs for their family; for instance, if their illness limits their ability to earn a salary or if they need to pay for their healthcare costs (Branca et al., 2019). Dealing with the diseases that livestock suffer from is an additional cost: the 2001 foot-and-mouth outbreak cost the UK taxpayer £3.1 billion (Thompson et al., 2002).

Due to livestock production being subsidised in many Western nations, the price of meat tends to be much cheaper than its true cost. After sugar and rice, animal products are the third most subsidised food groups in OECD countries, and subsidies for GHG emissions-intensive agricultural products like meat have risen since the early 1990s (Mamun et al., 2019). Earlier research shows that the European Union's Common Agricultural Policy import and export levies and subsidies on ruminant meat reduced international prices by 15% (Anderson and Tyers, 1984). Agricultural subsidies therefore lower food prices, which can increase consumption of these food products but may also reduce farmers' incomes in countries where subsidies are lacking, as their meat is outcompeted by artificially cheap meat produced elsewhere (Clapp, 2016).

When negative externalities are factored in, such as the cost of environmental and human health consequences of animal agriculture and meat consumption, the true cost of animal agriculture is much higher than what most consumers pay. If health care costs were included in the price of meat, processed meat would increase in cost by 25% on average (and by over 100% in high-income countries), and red meat costs would increase by 4% on average (up to 25% in high-income countries) (Springmann et al., 2018b). The global health care costs of only red and processed meat are estimated at $285 billion (Springmann et al., 2018b). Equally, if animal welfare costs were included in valuations, the true cost of animal agricultural production may be even larger (Carlier and Treich, 2020).

Wastage of meat has economic consequences too. In the UK, for example, approximately 28% of food thrown away in packaging that had not been opened was meat, fish, dairy and eggs are wasted by households each year compared with just 17% of fresh vegetables and salad (WRAP, 2014). The wastage for meat and fish alone equated to £2.1 billion in food bought but not consumed (WRAP, 2012). If we changed diets to meet recommended dietary guidelines, we would produce environmental benefits worth US $234 billion per year and would save US $735 billion a year in reduced health-related costs; these savings increase as more people switch to eating less meat and more plant-based diets (Springmann et al., 2016).

## 2 Interventions to reduce overconsumption of meat

It is now widely accepted that diets need to change to reduce meat overconsumption for the sake of human health and that of the planet. This aligns with the ‘One Health’ approach, which considers human, domesticated animal, wild animal and planetary health as interlinked. However, what is not yet known is the most effective ways to do this. We undertook an online survey to anonymously ask 50 sustainable diets experts (sent to members of the Food & Climate Research Network) what they thought were the most crucial knowledge gaps that need addressing to transition to more sustainable diets that reduce overconsumption of meat. The most frequently mentioned knowledge gaps were related to working out how to encourage consumers to buy more sustainable food, which were mentioned by half of the experts. We therefore focus the remainder of this article on effective interventions to transition to more sustainable diets that reduce overconsumption of meat and barriers that need to be overcome to address this.

**Fig. 1:**
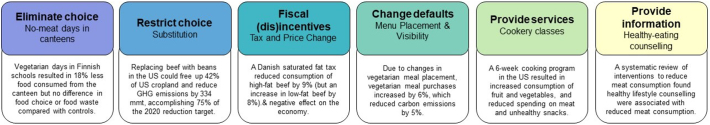
Examples of different intervention types to reduce meat overconsumption; darker colours represent potentially more effective but less feasible and socially acceptable options (based on data from Lombardini and Lankoski, 2013 (eliminate choice); Harwatt et al., 2017 (restrict choice); Bødker et al., 2015 (fiscal disincentives); Hansen et al., 2019 (change defaults); Flynn et al., 2013 (provide services); Diepeveen et al., 2013 (provide information)).

There are many options available for interventions, ranging from more controversial but potentially more effective measures that eliminate choice, such as restrictions on certain products, to more acceptable but somewhat less effective measures that provide information, such as awareness raising campaigns (Fig. 1). Like other public health interventions, dietary intervention effectiveness is highly context-specific, with barriers and enablers depending on local environments and socio-demographic variables. Care must be taken to ensure that interventions do not further widen dietary inequalities, with many already struggling to access healthy food. For food purchase and consumption interventions to be effective for the consumer to change their behaviour, they should address one or more of the following (based on Ranganathan et al., 2016):

1. **Minimise disruption**, for example, producing affordable, recognisable and tasty plant-based alternatives;
2. **Sell a compelling benefit**, such as improved taste, health benefits, or reduced cost;
3. **Maximise awareness**, which can be informed by traditional food marketing strategies such as putting plant-based foods at the top of a menu;
4. **Help shift norms** so that plant-based foods become the default rather than seen as a fringe behaviour.

## 3 Barriers to reducing overconsumption of meat

For consumers, accessing and choosing more sustainable foods that consist of less meat and more plants is not easy because the food system is inherently complex and embedded within deeper cultural, economic and political systems that are hard to change and often do not incentivise healthy, sustainable food consumption. The following factors are thought to contribute to continued unsustainable eating practices (adapted from Stubbs et al., 2018):

1. “**Choice architecture**”, where social factors such as traditions and cultural frames of reference persist - meat is not only a source of nourishment, but also a cultural symbol closely linked with identities and is highly politicised.
2. Most food choices are due to **ingrained habits** that are hard to change;
3. A **lack of consumer knowledge** of the relationship between food, environment and health – as a result, consumers do not have a clear frame of reference for what a sustainable diet is;
4. The **belief that meat is the best source of protein**;
5. Consumer **reluctance to learn about the negative impacts** of meat;
6. A **lack of prioritising sustainability** over taste, convenience and price;
7. Humans, in general, choose behaviours that have **short-term payoffs** and are less concerned with long-term costs, even towards their own health;
8. Healthy, environmentally friendly food can be **more expensive, rarer to find**, and/or take **longer to prepare**;
9. Government **subsidises and incentives** encourage unhealthy food commodities and environmentally damaging farming practices;
10. Unyielding **power of large food companies** to lobby governments and manipulate consumers;
11. **A sustained mantra that we can “innovate”** ourselves out of this mess by creating new agri-tech solutions to reduce environmental damages without considering that we need systemic, not just procedural, change;
12. **Lack of incentives** for food supply chain actors and consumers to change.

Overcoming these problems will likely be difficult and involve engaging with global leaders – both within and outside of governments – to aid dietary transitions away from overconsumption of meat.

## 4 The role of champions in changing diets to reduce overconsumption of meat

Leaders in nations where its citizens consume too much meat must begin to act on this issue to achieve planetary health. Considering the scale and nature of the problem, actions to reduce overconsumption of meat should take place along the entire supply chain. Changing consumer behaviour is crucial but, alone, is likely to be too slow and too small in scale to ensure a truly sustainable food system. To effect change at a systems level, interventions should make it easier for consumers to choose more sustainable food choices whilst also focusing on production, transportation and processing of food and agricultural products.

### 4.1 Fill the leadership gap

It is important that leaders of countries with high meat consumption embrace this challenge for the sake of their citizens' health and that of the planet's, but political action on aiding dietary transition away from overconsumption of meat has remained weak (Johnston et al., 2014; Prey, 2014). Some argue this is because politicians should not intervene in what citizens are eating, yet many countries have policies related to reductions in other unhealthy foods such as salt and sugar intake. In the UK, the Department for Environment, Farming and Rural Affairs commissioned a report in 2009 on sustainable diets and the recommendations called for a reduction in meat to achieve better environmental and health outcomes. Despite this finding, the UK Government's former Environment Secretary, Rt Hon Michael Gove, later told farmers in 2018 that meat is crucial for a balanced diet and, that same year in an interview with the *Guardian* newspaper said “it's not my job to micro-manage what goes into a shopping basket”. There has since been a sustained lack of UK governmental action on addressing this issue (notwithstanding a new UK Food Strategy in development). Sustainable diet advocates clearly have a long way to go to conveying policymakers of the business case for why we need to reduce overconsumption of meat.

### 4.2 Subsidize for ‘One Health’

Policymakers could also alter the agricultural subsidy and incentive system to ensure we are not using taxpayers' money to subsidize environmentally damaging behaviours that encourage production of unhealthy foods (Simon, 2013). Removal of harmful subsidies and replacing them with positive incentives could be one way of addressing this. As one example, the inherent inefficiency of feeding human-edible crops to livestock could be reduced by redirecting subsidies and policies towards producing nutritious, sustainable crops (such as legumes) for direct human consumption. Farmers are key players in the food system and must be supported through governmental and industry incentives to use more sustainable practices. Whilst we do not suggest that permanent pasture should be converted to arable land due to the carbon emissions associated with this change, arable farmers could instead be encouraged to grow foods directly for human consumption rather than for livestock feed. Legumes, in particular, should be incentivised, given their high protein content and nitrogen-fixing abilities, thereby reducing the need for nitrogen fertiliser.

### 4.3 ‘One Health’ nutritional training

Medical professions in developed nations are becoming more aware of the joint human and environmental health consequences of meat-rich diets, though additional sustainable dietary training for General Practitioners (who are the first point of call for most patients seeking medical help) is advised. It is imperative that, as part of the prevention and treatment of lifestyle-related diseases, patients are educated and appropriately referred to multidisciplinary prescribers of optimal nutrition and other determinants of wellbeing. Training of this ‘One Health’ approach should be expanded for all medical professionals who advise on health and diets.

### 4.4 Partnering with the food industry

The food industry should also be incentivised to take responsibility for systems change. Important progress is already being made in some areas, such as restrictions on advertising junk food and, in the UK in particular, there has been a widespread change recently with leading supermarkets and food outlets offering substantially more plant-based options than in the past. Providing more choice of plant-based foods can help people shift to reduced meat diets: one recent study found that doubling the proportion of vegetarian foods offered in a canteen increased vegetarian sales by between 41% and 79% (Garnett et al., 2019). Food sustainability experts and policymakers need to work more closely with the food industry to ensure these supply chains are working towards social, economic and environmental sustainability, and government intervention should encourage this. Collaborations with the third sector could assist here; already, some partnerships are bearing fruit. For instance, WWF has been working with the food catering firm Sodexo, which has resulted in the company launching a new menu (Green & Lean) that features meals consisting of two-thirds plants, grains and pulses. Additional partnerships like this could prove extremely effective.

## 5 Conclusions

In light of the climate emergency and the rapidly increasing double burden of malnutrition, there has arguably never been a more important time in human history to transform our food system for the sake of humans and nature. In this paper, we explored the impact of the overconsumption of meat on the three pillars of sustainability; social (including health), environmental and economic. We note a range of interventions available to transform the food system to more sustainable diets that reduce overconsumption of meat. However, these interventions are highly context-specific, and are likely to vary in their effectiveness, since there are a wide range of complex barriers which exist. Addressing this challenge is therefore difficult and requires action across the whole supply chain, including changing consumer behaviour. More interdisciplinary thinking is required, whereby sustainable food system researchers and practitioners must work together and collaborate with the food and agricultural industries, health and social services, educational institutes as well as policymakers, media and civil society. Complex problems such as overconsumption of meat require complex solutions, but together we can - and must – urgently work towards a healthy food system for people and the planet.

## Declaration of competing interest

The authors declare that they have no known competing financial interests or personal relationships that could have appeared to influence the work reported in this paper.
